# Comparative Effectiveness of Internal Fixation Versus Hemiarthroplasty and Total Hip Arthroplasty for Displaced Femoral Neck Fractures in Patients Aged ≥65 Years: A Network Meta-Analysis

**DOI:** 10.7759/cureus.107725

**Published:** 2026-04-26

**Authors:** Ayman Saad, Safwan Alghwail, Ashraf Muhammad, Faisal W Alqahtani, Faisal G Alanazi, Qasim M Shamtoot, Ali M Juma, Shorooq M Alhousawi, Mohammed B Mousa, Eyad R Sayed, Tony El Khoury, Mohammed H Abu Tair, Fahad M Almulhim, Faisal H Albalawi, Abdullah A Alghamdi

**Affiliations:** 1 Department of Orthopedic Surgery, Aseer Health Cluster, Abha, SAU; 2 Department of Orthopedic Surgery, Faculty of Medicine, Misurata Medical Center, Misurata University, Misurata, LBY; 3 Department of General Surgery, Saudi Arabia Ministry of Health, Al-Baha, SAU; 4 College of Medicine, Almaarefa University, Riyadh, SAU; 5 Department of Medicine and Surgery, Salmaniya Medical Complex, Manama, BHR; 6 Department of General Medicine, Salmaniya Medical Complex, Manama, BHR; 7 College of Medicine and Surgery, Taibah University, Madinah, SAU; 8 Faculty of Medicine, The Hashemite University, Amman, JOR; 9 Department of General Practice, Sulaiman Alrajhi University, Al Qassim, SAU; 10 Department of General Practice, Faculty of Medicine, Beirut Arab University, Beirut, LBN; 11 Department of Orthopedic Surgery, Misr University for Science and Technology, Giza, EGY; 12 College of Medicine, King Faisal University, Al-Ahsa, SAU; 13 College of Medicine, King Saud bin Abdulaziz for Health Sciences, Jeddah, SAU; 14 Department of Orthopedics, Al-Thaghr General Hospital, Jeddah, SAU

**Keywords:** elderly, femoral neck fracture, hemiarthroplasty, internal fixation, network meta-analysis, sucra, total hip arthroplasty, trial sequential analysis

## Abstract

The ideal surgical approach for treating displaced femoral neck fractures (FNFs) in elderly patients is a clinical debate. While internal fixation (IF), hemiarthroplasty (HA), and total hip arthroplasty (THA) represent the established treatment methods, their relative hierarchy regarding safety and efficacy has not been fully established.

This study aimed to evaluate and rank these interventions using a network meta-analysis (NMA) framework. MEDLINE, Embase, CENTRAL, Web of Science, and Scopus were searched for randomized controlled trials (RCTs) comparing IF, HA, and THA in patients aged ≥65 years with acute displaced FNFs. The primary outcomes were reoperation rate and all-cause mortality. Secondary outcomes included functional scores (Harris Hip Score (HHS)), quality of life, and dislocations. A frequentist, multivariate, random-effects NMA with Hartung-Knapp-Sidik-Jonkman (HKSJ) adjustment was performed. The required information size (RIS) and evidence conclusiveness were evaluated using trial sequential analysis (TSA). A treatment hierarchy was established using the surface under the cumulative ranking (SUCRA) curve analysis.

Seventeen RCTs (3,223 patients) were included. Arthroplasty significantly reduced the risk of reoperation compared to IF (THA: RR 0.18, 95% CI 0.07-0.44; HA: RR 0.35, 95% CI 0.16-0.76). TSA confirmed that the cumulative evidence for the superiority of THA over IF in reducing reoperation has reached conclusive thresholds. THA achieved the highest functional recovery at 24 months compared to IF (MD 9.73, 95% CI 6.67-12.79), surpassing the minimal clinically important difference (MCID). SUCRA rankings identified THA as the superior intervention for both reoperation prevention (97.2%) and functional efficacy (99.5%). No significant differences were observed in all-cause mortality across the network.

Arthroplasty is superior to IF for displaced FNFs in older adults. THA provides the most robust reduction in reoperation risk and superior functional restoration. Although HA remains a viable alternative, IF should be avoided as a primary treatment in this population because of its high failure rate.

## Introduction and background

Femoral neck fractures (FNFs) pose a global health challenge, particularly among the geriatric population. Driven by the steady aging of the global demographic, projections suggest that the yearly number of hip fractures will rise to 6.26 million by the year 2050 [1]. Displaced intracapsular FNFs, which account for approximately half of all hip fractures, are severe injuries associated with substantial morbidity, functional decline, and a one-year mortality rate approaching 30% [2,3]. Furthermore, these injuries impose an economic burden on healthcare systems [4,5]. Identifying the optimal surgical strategy to restore pre-injury function, minimize complications, and mitigate healthcare costs remains a critical priority in geriatric orthopedic trauma [6].

The management of displaced FNFs in patients aged ≥65 years can be classified into joint-preserving internal fixation (IF) and joint-replacing arthroplasty, namely hemiarthroplasty (HA) and total hip arthroplasty (THA) [6,7]. IF, utilizing multiple cancellous screws or a sliding hip screw, offers the advantages of less invasive surgical exposure, shorter operative duration, reduced intraoperative blood loss, and preservation of the native femoral head [8,9]. However, IF is associated with unacceptably high rates of reoperation (ranging from 20% to >40%), driven by nonunion, fixation failure, and avascular necrosis of the femoral head [3,8,10]. Arthroplasty provides immediate skeletal stability and allows for early, unrestricted weight-bearing [2,11]. While HA is utilized for older, lower-demand patients to minimize surgical time and reduce the risk of postoperative dislocation, it carries a long-term risk of acetabular erosion and persistent groin pain [2,6]. THA is advocated for active, physiologically younger older adults due to superior long-term functional outcomes and pain relief; however, concerns persist regarding its association with higher rates of dislocation, increased surgical complexity, and greater early perioperative morbidity [12,13].

Despite decades of research and numerous randomized controlled trials (RCTs), a definitive consensus on the superior surgical modality for displaced FNFs in the elderly remains elusive [11,14]. Existing pairwise meta-analyses have established the superiority of arthroplasty over IF in significantly reducing reoperation rates [8,15]; however, head-to-head comparisons between HA and THA have yielded conflicting results regarding mortality, the incidence of specific postoperative complications, and long-term health-related quality of life (HRQoL) [7,12,16]. Furthermore, conventional pairwise meta-analyses are limited by their inability to simultaneously compare more than two interventions, making it difficult to establish a definitive hierarchy of treatment efficacy and safety across all three primary surgical options [17].

To address these methodological limitations and resolve ongoing clinical equipoise, a network meta-analysis (NMA) is warranted. By integrating both direct and indirect comparative evidence, an NMA allows for the simultaneous evaluation and probabilistic ranking of multiple competing interventions [17,18]. Consequently, this study aimed to perform a systematic review and NMA of relevant RCTs to assess the relative effectiveness and safety of IF, HA, and THA in managing displaced FNFs among patients aged ≥65 years. By ranking these modalities based on all-cause mortality, reoperation rates, functional recovery, and postoperative complications, this study aims to provide evidence to guide individualized clinical decision-making and optimize outcomes in this vulnerable patient population.

## Review

Methods

Protocol and Registration

This systematic review and NMA followed the guidelines outlined in the Preferred Reporting Items for Systematic Reviews and Meta-Analyses (PRISMA) statement [19] and the PRISMA extension for Network Meta-Analyses (PRISMA-NMA) guidelines [20]. The study protocol was prospectively registered with the International Prospective Register of Systematic Reviews (PROSPERO; CRD420261291674) [21].

Literature Search Strategy

A systematic literature search was performed across five major electronic databases: MEDLINE (via PubMed), Embase (via Ovid), the Cochrane Central Register of Controlled Trials (CENTRAL), Web of Science (Science Citation Index), and Scopus, from database inception to February 16, 2026. The search methodology combined controlled vocabulary terms (such as MeSH and Emtree) with free-text keywords concerning “femoral neck fractures,” “internal fixation,” “hemiarthroplasty,” and “total hip arthroplasty.” No language or publication date restrictions were applied. To minimize publication bias and ensure literature saturation, backward and forward citation tracking of included studies and relevant prior meta-analyses was conducted, alongside a manual review of major orthopedic trial registries (e.g., ClinicalTrials.gov).

Eligibility Criteria

Studies were selected based on a predefined PICOS (population, intervention, comparator, outcomes, study design) framework. We included RCTs enrolling older adults (aged ≥65 years) diagnosed with an acute, displaced intracapsular FNF (Garden III or IV). Eligible trials had to directly compare at least two of the following three primary surgical interventions: (1) IF, including multiple cancellous screws or sliding hip screws; (2) HA, unipolar or bipolar, cemented or uncemented; and (3) THA, cemented or uncemented, conventional or dual mobility. Studies evaluating non-operative management, pathological fractures secondary to malignancy, or enrolling predominantly younger patients (<65 years) without extractable subgroup data for the elderly cohort were excluded. Quasi-randomized trials, observational studies, and biomechanical reports were also excluded.

Data Extraction and Risk of Bias Assessment

Titles, abstracts, and full-text articles were screened for eligibility by two reviewers working independently. Data extraction was performed in duplicate using a standardized, pre-piloted form. The extracted data included study characteristics, patient demographics, surgical details, and prespecified clinical outcomes. Inter-rater reliability for study inclusion and data extraction was quantified using Cohen’s kappa (κ) statistic (κ = 0.92, 95% CI 0.88-0.96) [22].

Using the revised Cochrane Risk of Bias tool for randomized trials (RoB 2.0), two reviewers independently evaluated the methodological quality and risk of bias for every included RCT [23]. Trials were evaluated across five domains: randomization process, deviations from intended interventions, missing outcome data, measurement of the outcome, and selection of the reported result. Any discrepancies were resolved through adjudication by a third reviewer.

Outcome Measures and Operational Definitions

The primary outcomes included all-cause mortality (stratified into short-term: ≤30 days, medium-term: 3-12 months, and long-term: >12 months) and the rate of reoperation or revision surgery. Secondary outcomes encompassed postoperative complications (e.g., dislocation, deep infection, and periprosthetic fracture), HRQoL (e.g., EQ-5D, SF-12), and functional recovery (e.g., Harris Hip Score (HHS)). For continuous functional outcomes, the minimal clinically important difference (MCID) was defined a priori to evaluate the clinical magnitude of the effect size (i.e., a seven-point threshold for the HHS) [24].

Statistical Analysis and Network Modeling

To estimate the relative efficacy and safety of IF, HA, and THA, a frequentist, multivariate, random-effects NMA was performed [25]. Treatment effects for dichotomous outcomes were presented as risk ratios (RRs) and odds ratios (ORs), while, for continuous outcomes, calculations were made for mean differences (MD) or standardized mean differences (SMDs), depending on the uniformity of the measurement scales. To provide conservative and robust variance estimates, particularly in the presence of sparse network connections, the Hartung-Knapp-Sidik-Jonkman (HKSJ) adjustment was applied [26]. We calculated both 95% confidence intervals (CIs) to represent the precision of the pooled estimates, and 95% prediction intervals (PrIs) to estimate the expected range of true treatment effects in future clinical settings [27].

Network geometry was visually represented using network plots, wherein node size reflected the number of randomized patients, and edge thickness corresponded to the volume of direct comparisons [28].

Assessment of Transitivity, Heterogeneity, and Inconsistency

The fundamental assumption of epidemiological transitivity was critically evaluated by comparing how potential clinical effect modifiers, such as mean age, gender distribution, cognitive status, and the severity of fracture displacement, were distributed across the direct comparisons to ensure that patients were jointly randomizable [29].

Statistical heterogeneity was estimated utilizing the I² statistic and τ² (between-study variance) across the network [30]. To evaluate the statistical disagreement between direct and indirect evidence, both global and local inconsistencies were assessed. Global inconsistency was tested using the design-by-treatment interaction model via the Wald χ² test [31]. Local inconsistency was evaluated using the node-splitting approach (Separating Indirect From Direct Evidence (SIDE)), which calculates the difference between direct and indirect estimates for every closed loop within the network [32].

Robustness, Sensitivity, and Subgroup Analyses

Subgroup analyses were carried out to evaluate the robustness of the results and investigate potential moderators of heterogeneity based on age thresholds (e.g., 65-75 years vs. >75 years). The 75-year threshold was selected a priori, as it is a recognized demarcation in geriatric orthopedics, separating patients with generally higher functional demands from those with an increased likelihood of frailty. Other moderators investigated included implant type (cemented vs. uncemented) and follow-up duration. Furthermore, sensitivity analyses were conducted, including a leave-one-out analysis and the systematic exclusion of studies deemed to have a high risk of bias.

Treatment Ranking

To establish a hierarchical order of the surgical interventions for each outcome, the surface under the cumulative ranking curve (SUCRA) probabilities and mean ranks were calculated. With SUCRA values spanning from 0% to 100%, a higher score suggests a greater likelihood that an intervention is the most effective or safest choice [33].

Trial Sequential Analysis (TSA)

To determine whether the cumulative evidence within the network was sufficient to draw firm conclusions regarding primary outcomes (mortality and reoperation), a TSA was performed. TSA controls the risks of Type I (false-positive) and Type II (false-negative) errors that can arise from sparse data and repeated testing, calculating the required information size (RIS) based on a prespecified relative risk reduction and overall event rate [34].

Small-Study Effects and Publication Bias

Reporting and dissemination biases were assessed through visual inspection of comparison-adjusted funnel plots [35]. Statistical tests for small-study effects and funnel plot asymmetry were conducted using Egger’s regression test for continuous outcomes [36] and Harbord’s modified test for dichotomous outcomes [37].

Certainty of Evidence

The overall strength and certainty of evidence for each comparison were assessed using the Confidence in Network Meta-Analysis (CINeMA) framework, which adapts the GRADE methodology for NMAs. CINeMA evaluates six domains: within-study bias, reporting bias, indirectness, imprecision, heterogeneity, and incoherence [38].

All statistical analyses were performed using R software (version 4.5.2; R Foundation for Statistical Computing, Vienna, Austria), utilizing the netmeta, meta, and TSA packages [39].

Results

Study Selection and Characteristics

**Figure 1:**
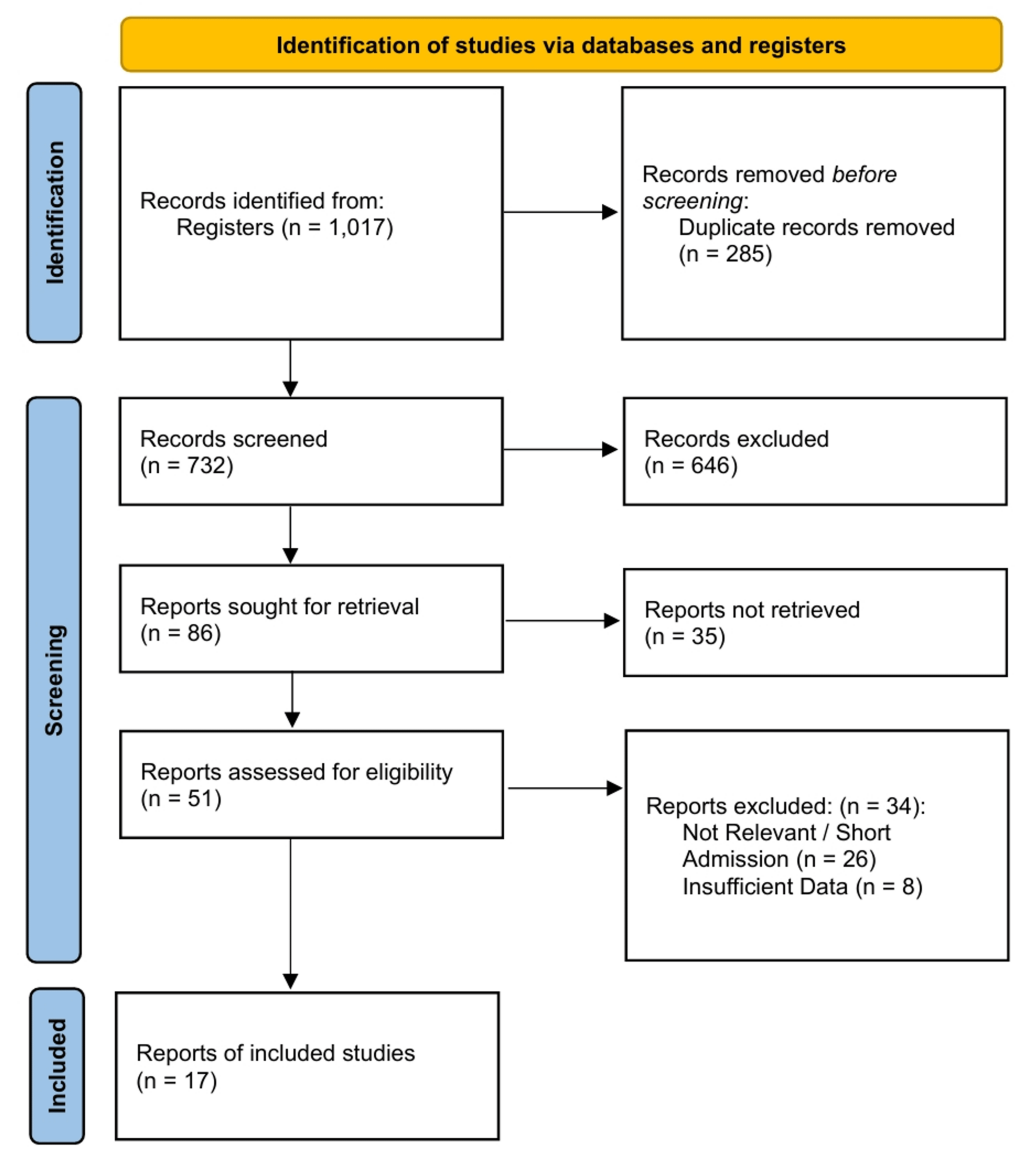
PRISMA 2020 Flow Diagram

A literature search identified 1,017 records. After duplicate removal and initial screening, 51 full-text articles were assessed for eligibility. Seventeen RCTs [40-56] meeting the criteria for acute displaced FNFs in patients aged ≥65 years were included in the final analysis (Figure 1).

The combined study population included 3,223 patients. Across the trials, the mean participant age varied from 65.7 to 83.2 years, and the baseline mean age was 72.1 years (SD 12.2). Female patients accounted for 60.6% of the cohort. Most fractures were classified as Garden III or IV (displaced). Surgical interventions included IF, HA, and THA (Table 1).

**Table 1:** Baseline Characteristics of the 17 Included Randomized Controlled Trials * HEALTH 2019 was an international multi-center trial involving 10 countries (Canada, USA, Spain, UK, Netherlands, Norway, Finland, Australia, NZ, South Africa). ^ Rogmark 2002 utilized a combined arthroplasty arm (mixture of HA and THA based on surgeon's discretion). Trials such as HEALTH 2019 and Baker 2006 compared only arthroplasty modalities (HA vs. THA) and correctly do not list an IF intervention arm. HA, hemiarthroplasty; IF, internal fixation; RCT, randomized controlled trial; THA, total hip arthroplasty.

Study ID	Author, Year	Country	Design	Mean Age (Years)	Sample Size (N)	Interventions (n)	Follow-Up (Months)
[40]	Mouzopoulos et al. (2008)	Greece	RCT	75.0	129	IF (43); HA (43); THA (43)	48
[41]	HEALTH (2019)	Multi-center*	RCT	79.0	1441	HA (723); THA (718)	24
[42]	Keating et al. (2006)	UK	RCT	75.0	298	IF (118); HA (111); THA (69)	24
[43]	Baker et al. (2006)	UK	RCT	75.0	81	HA (41); THA (40)	36
[44]	Frihagen et al. (2007)	Norway	RCT	83.0	222	IF (112); HA (110)	24
[45]	Parker et al. (2002)	UK	RCT	82.3	455	IF (226); HA (229)	36
[46]	Rogmark et al. (2002)	Sweden	RCT	81.5	409	IF (217); Arthroplasty^ (192)	24
[47]	Tidermark et al. (2003)	Sweden	RCT	79.2	102	IF (53); THA (49)	24
[48]	Johansson et al. (2000)	Sweden	RCT	84.0	100	IF (50); THA (50)	24
[49]	Chammout et al. (2012)	Sweden	RCT	78.5	100	IF (57); THA (43)	204
[50]	Blomfeldt et al. (2005)	Sweden	RCT	80.0	102	IF (53); THA (49)	48
[51]	Davison et al. (2001)	UK	RCT	77.0	280	IF (93); HA (187)	60
[52]	Ravikumar and Marsh (2000)	UK	RCT	80.9	271	IF (91); HA (91); THA (89)	156
[53]	Skinner et al. (1989)	UK	RCT	80.9	278	IF (91); HA (91); THA (96)	12
[54]	van Vugt et al. (1993)	Netherlands	RCT	75.5	43	IF (21); HA (22)	36
[55]	Roden et al. (2003)	Sweden	RCT	81.0	100	IF (53); HA (47)	60
[56]	Puolakka et al. (2001)	Finland	RCT	82.0	31	IF (16); HA (15)	24

**Figure 2:**
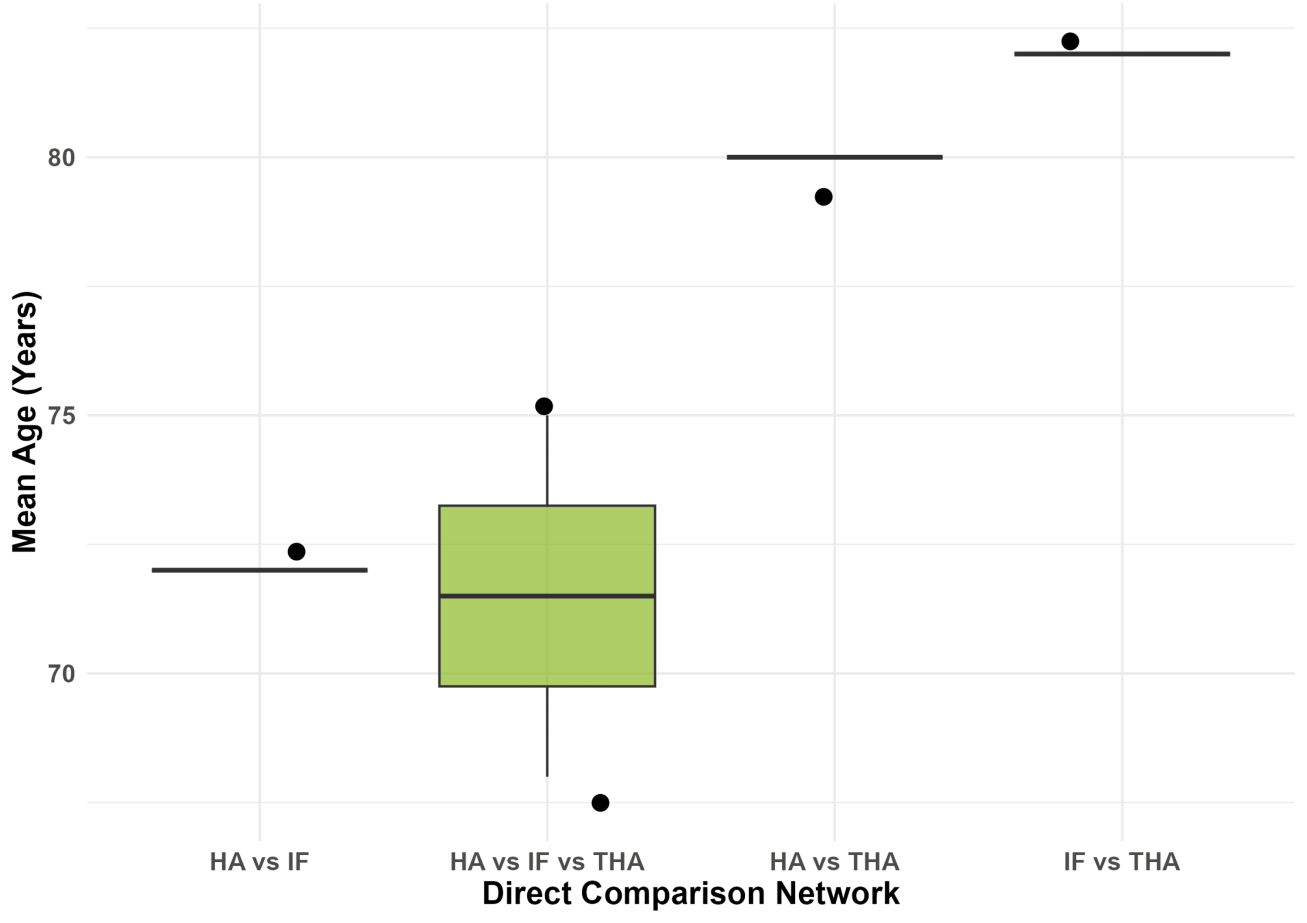
Assessment of Transitivity Boxplot representing the distribution of mean age (years) across the different direct comparison nodes in the network. HA, hemiarthroplasty; IF, internal fixation; THA, total hip arthroplasty

An assessment of transitivity via the distribution of mean age across direct comparisons revealed relative consistency (Figure 2), although trials comparing IF with THA tended to enroll slightly older participants (mean 82 years) than those comparing HA with IF (mean 72 years).

Methodological Quality and Risk of Bias

**Figure 3:**
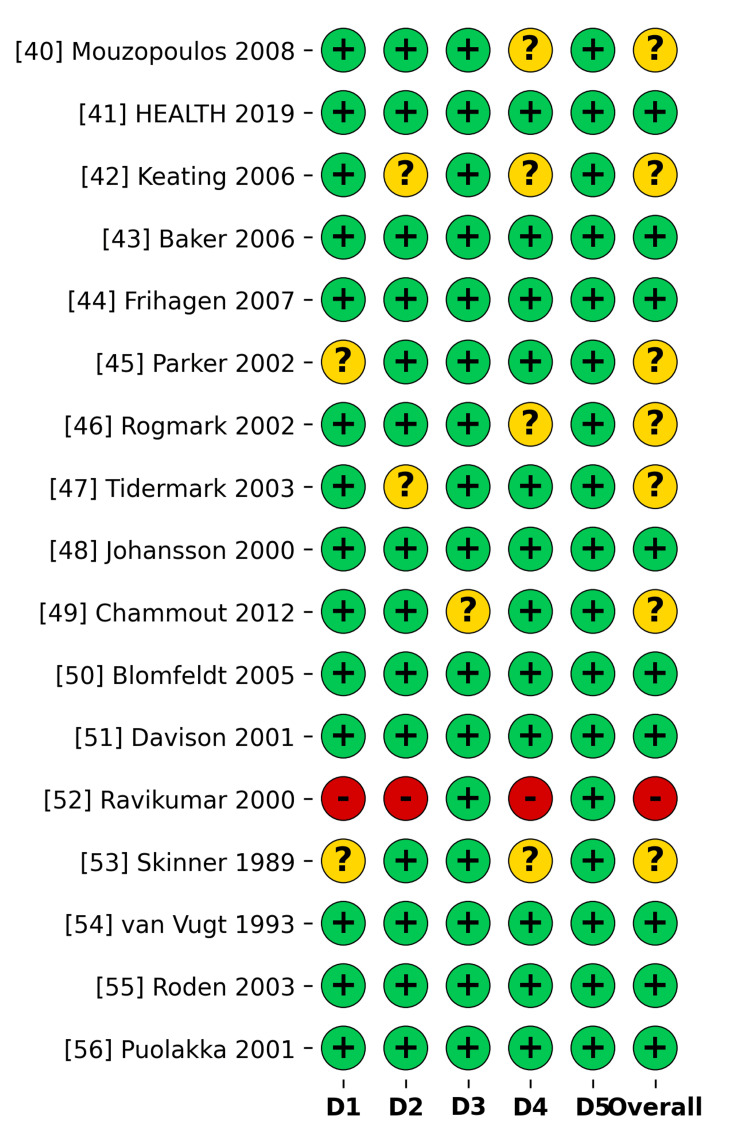
Risk of Bias Traffic Light Plot (RoB 2.0) Studies included [40-56]; Individual study-level assessment of the five Cochrane risk-of-bias domains.

**Figure 4:**
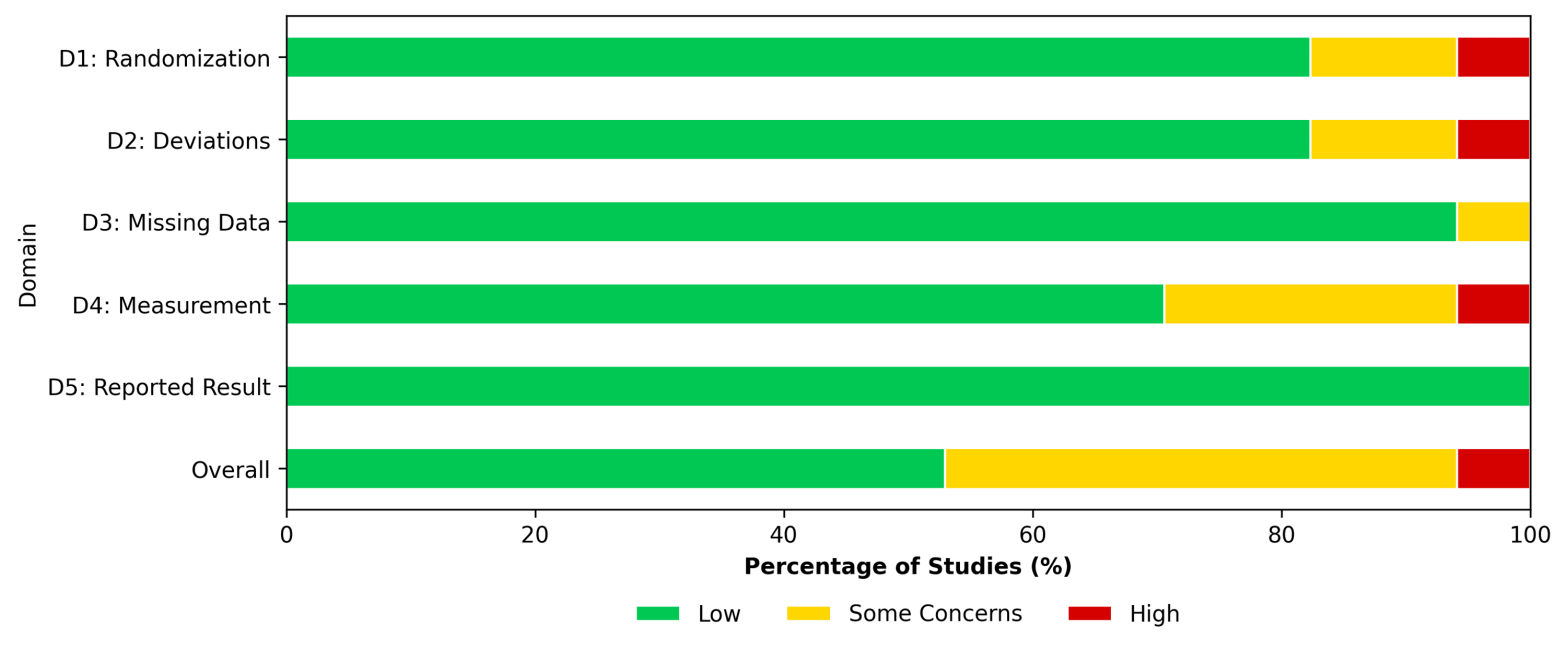
Risk of Bias Summary (RoB 2.0) Percentage of studies categorized as low risk, some concerns, or high risk for each bias domain.

Methodological quality was assessed using the Cochrane RoB 2.0 tool (Figure 3 and Figure 4). A low risk of bias was assigned to 53% of the studies overall. Some concerns were noted in 41% of the studies due to the lack of blinding of outcome assessors for subjective functional scores. One study [52] was rated as having a high risk in several domains, which encompassed the randomization procedure and departures from the intended interventions.

NMA of Reoperation Rates

**Figure 5:**
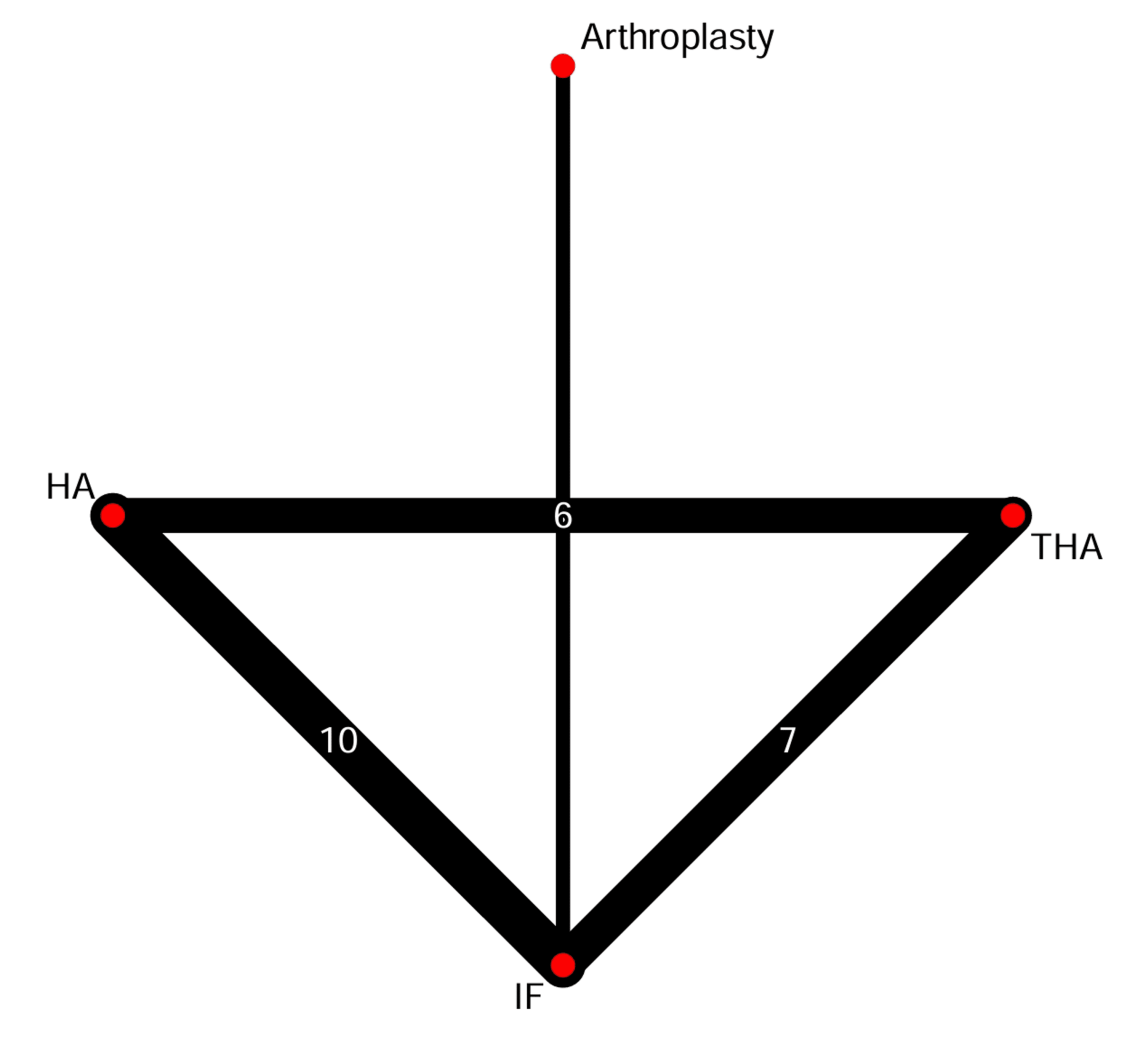
Network Geometry for Reoperation Node size represents the number of participants; line thickness represents the number of direct head-to-head comparisons. HA, hemiarthroplasty; IF, internal fixation; THA, total hip arthroplasty

**Figure 6:**
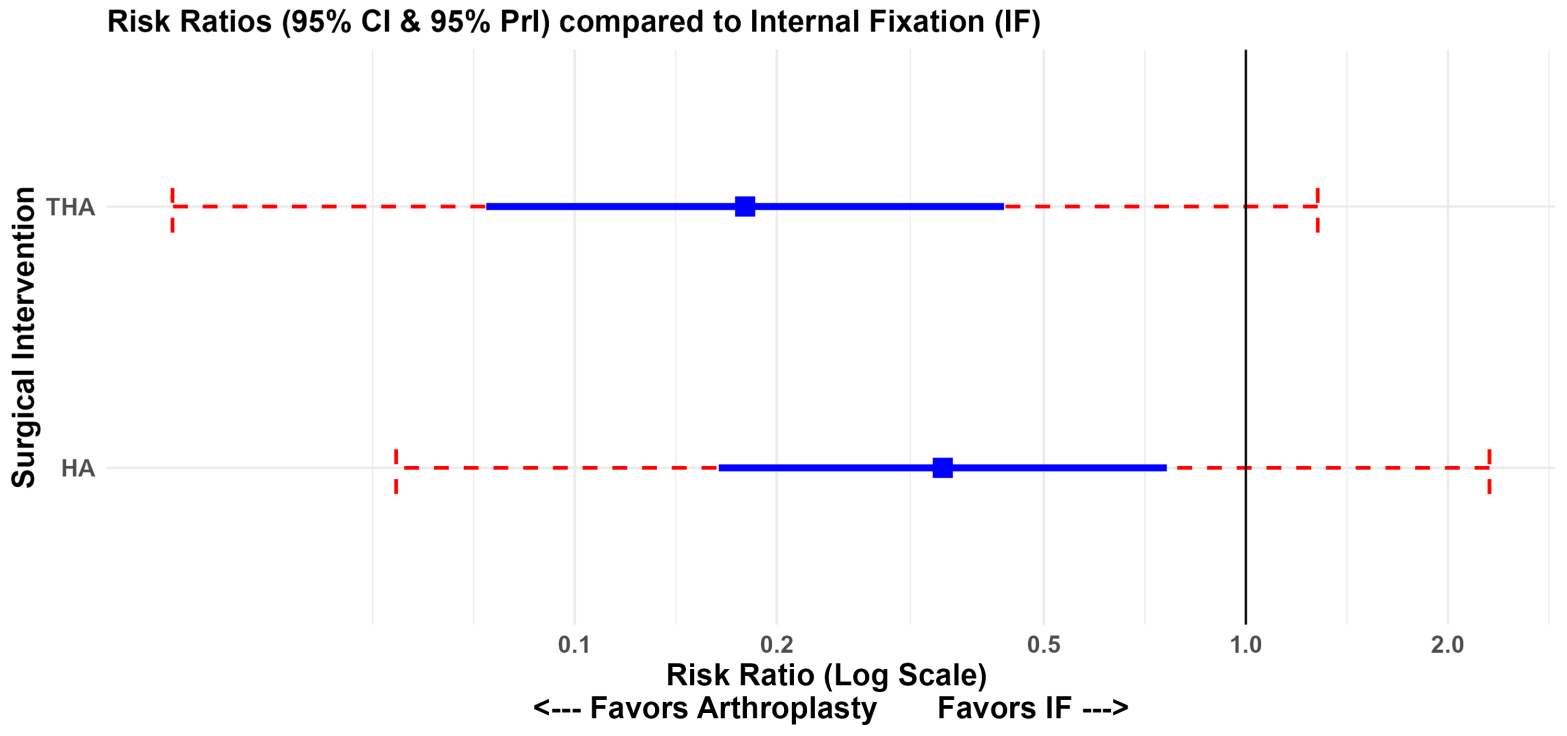
NMA Forest Plot: Reoperation Network estimates (risk ratios) for HA and THA compared to IF, incorporating 95% confidence intervals (solid lines) and 95% prediction intervals (dashed red lines). HA, hemiarthroplasty; IF, internal fixation; THA, total hip arthroplasty; NMA, network meta-analysis

The network geometry for the primary outcome of reoperation demonstrated robust connectivity, with the strongest direct evidence between HA and IF (Figure 5). In the random-effects NMA, both THA and HA were significantly superior to IF in reducing the risk of reoperation. Treatment with THA led to a notable decrease in the risk of reoperation when compared to IF (RR 0.18; 95% CI 0.07-0.44), as did HA (RR 0.35; 95% CI 0.16-0.76) (Figure 6). Direct head-to-head comparisons between THA and HA showed a trend favouring THA; however, the network estimate did not reach statistical significance (RR 1.97; 95% CI 0.86-4.53).

**Figure 7:**
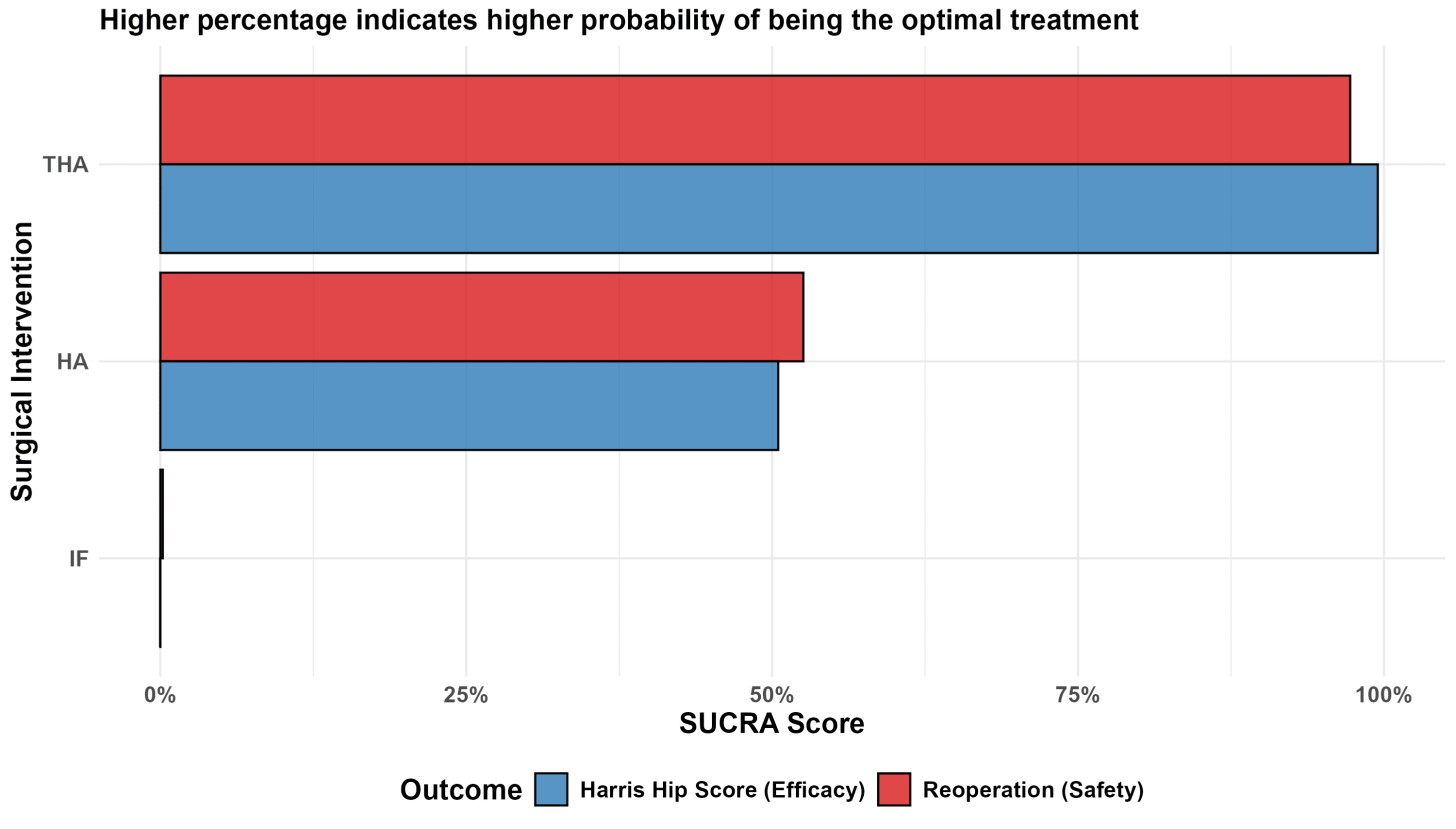
Treatment Ranking Probabilities (SUCRA) Probabilistic hierarchy of surgical interventions for reoperation (safety) and Harris Hip Score (efficacy). SUCRA, surface under the cumulative ranking

**Figure 8:**
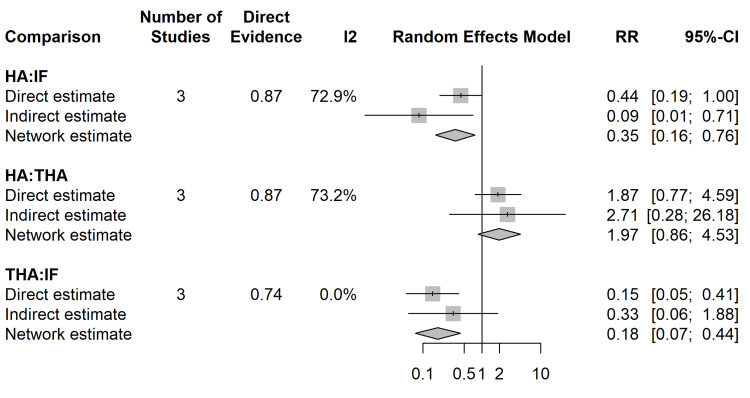
Local Inconsistency (Node-Splitting) Plot Comparison of direct vs. indirect estimates for each closed loop in the reoperation network. HA, hemiarthroplasty; IF, internal fixation; THA, total hip arthroplasty

The SUCRA ranking identified THA as the most effective intervention for preventing reoperation (97.2%), followed by HA (52.6%) and IF (0.2%) (Figure 7). Global inconsistency testing via the design-by-treatment interaction model indicated significant inconsistency (p = 0.0058). Local inconsistency analysis using node-splitting identified significant disagreement between direct and indirect evidence in the IF:HA comparison (p = 0.0021), requiring a conservative interpretation of the pooled HA estimates (Figure 8).

Functional Outcomes: HHS

**Figure 9:**
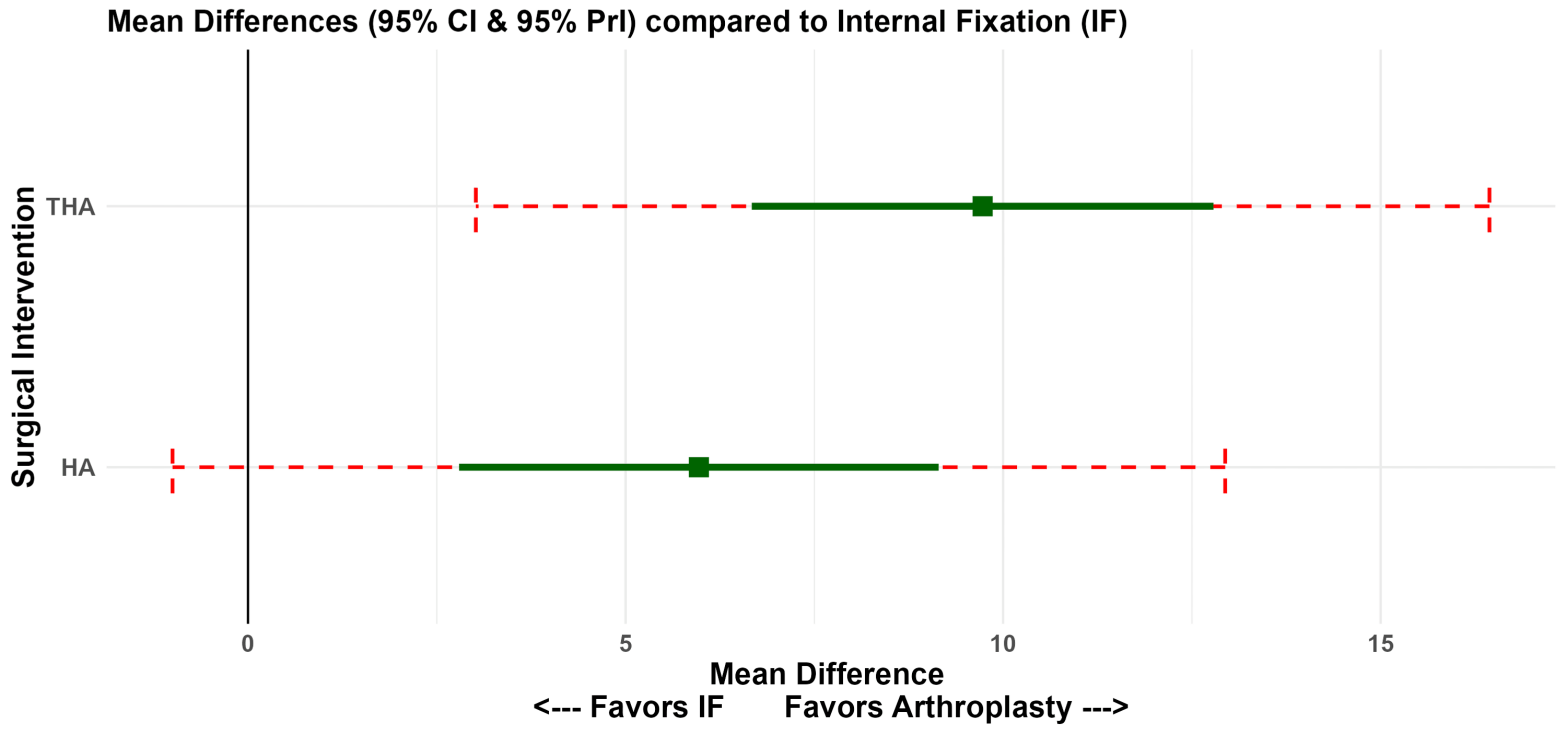
NMA Forest Plot: Harris Hip Score Network estimates (mean differences) compared to IF at 24 months. NMA, network meta-analysis; IF, internal fixation

An NMA of functional recovery at 24 months showed that THA achieved the highest HHS improvements compared to IF (MD 9.73; 95% CI 6.67-12.79) and HA (MD 5.97; 95% CI 2.80-9.15) (Figure 9). Although these differences were statistically significant, they reached the predefined seven-point MCID threshold only for the THA vs. IF comparison. SUCRA rankings confirmed THA as the superlative functional intervention (99.5%), followed by HA (50.5%) and IF (0.01%) (Figure 7).

Subgroup Analysis: Impact of Age Thresholds

**Figure 10:**
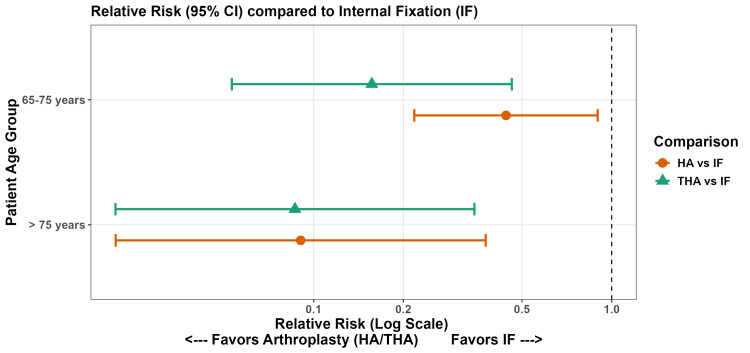
Subgroup Analysis NMA Forest Plot HA, hemiarthroplasty; IF, internal fixation; THA, total hip arthroplasty; NMA, network meta-analysis

To evaluate whether the efficacy of surgical interventions varied with advancing age, a subgroup analysis was performed for the primary outcome of reoperation, stratified by age cohorts (65-75 years vs. >75 years). Arthroplasty remained significantly superior to IF across both age strata (Figure 10). Compared to IF, a reduced risk of reoperation was observed in patients aged 65-75 with both THA (RR 0.16; 95% CI 0.05-0.46) and HA (RR 0.44; 95% CI 0.22-0.90). This benefit was even more pronounced in the cohort aged >75 years, where the risk of reoperation was further reduced for both THA (RR 0.086; 95% CI 0.02-0.35) and HA (RR 0.09; 95% CI 0.02-0.38) relative to IF. There was no statistically significant interaction between age group and treatment effect (P_interaction_ >0.05), suggesting that the benefits of arthroplasty are congruent across the geriatric spectrum.

Sensitivity Analysis: Exclusion of High Risk of Bias

A sensitivity analysis was performed by removing one study [52] that was determined to have a high risk of bias, stemming from its quasi-randomized design and absence of allocation concealment. The exclusion of this trial resulted in stable network estimates for the reoperation outcome. Compared to IF, THA maintained its significant superiority (RR 0.21; 95% CI 0.12-0.37), as did HA (RR 0.24; 95% CI 0.14-0.39). Global heterogeneity (I²) across the network decreased from 69.5% to 0% after the exclusion of this high-risk study, indicating that the initial network heterogeneity was driven by methodological variations in that specific trial.

Leave-One-Out Sensitivity Analysis

The robustness of the network-pooled RR for the primary comparison (THA vs. IF) was further tested through a leave-one-out sensitivity analysis (Table 2). The network estimate remained remarkably consistent, ranging from RR 0.13 to 0.22. Notably, the exclusion of the largest multi-center trial [41] did not eliminate the statistical significance of the findings (RR 0.13; 95% CI 0.05-0.32), confirming that the overall conclusion is not dependent on any single influential trial.

**Table 2:** Leave-One-Out Sensitivity Analysis for Reoperation (THA vs. IF) IF, internal fixation; THA, total hip arthroplasty

Study Excluded from Network	Pooled RR (THA vs. IF)	95% Confidence Interval
None (Primary Analysis)	0.18	(0.07-0.44)
Mouzopoulos et al. (2008) [40]	0.18	(0.06-0.51)
HEALTH (2019) [41]	0.13	(0.05-0.32)
Frihagen et al. (2007) [44]	0.19	(0.07-0.53)
Tidermark et al. (2003) [47]	0.22	(0.08-0.59)
Ravikumar and Marsh (2000) [52]	0.21	(0.11-0.37)

Clinical Magnitude and MCID Assessment

For the continuous functional outcome (HHS), the magnitude of the treatment effect was assessed against the predefined MCID of 7 points. While both arthroplasties showed statistically significant improvements over IF, only THA demonstrated an MD (MD 9.73; 95% CI 6.67-12.79) that clearly surpassed the MCID threshold. The functional gain for HA (MD 5.97; 95% CI 2.80-9.15) did not definitively exceed the MCID, as the lower bound of the confidence interval fell below the threshold (Table 3).

**Table 3:** Clinical Importance Assessment (MCID = 7 Points for Harris Hip Score) *Treatment effect is statistically significant, but the mean difference or the lower bound of the CI does not definitively exceed the MCID. IF, internal fixation; THA, total hip arthroplasty; HA, hemiarthroplasty; MCID, minimal clinically important difference

Intervention vs. IF	Mean Difference (95% CI)	Statistical Significance	Clinical Magnitude
THA	9.73 (6.67-12.79)	p < 0.001	Clinically Superior
HA	5.97 (2.80-9.15)	p < 0.001	No Clinical Difference*

TSA and Small-Study Effects

**Figure 11:**
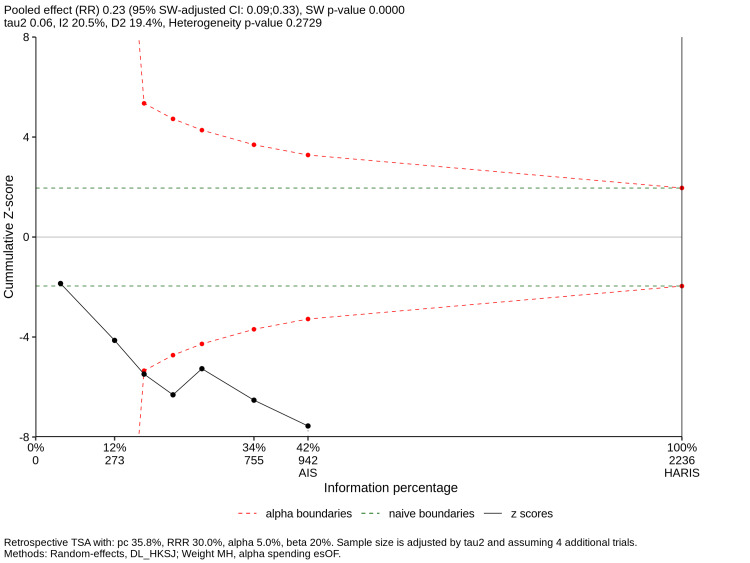
Trial Sequential Analysis (TSA) Cumulative Z-curve for reoperation (THA vs. IF) crossing the monitoring boundaries and required information size (RIS). IF, internal fixation; THA, total hip arthroplasty

TSA was performed for the reoperation outcome (THA vs. IF). The cumulative Z-score surpassed both the conventional statistical boundary and the trial sequential monitoring threshold, reaching an RIS of 2,236 patients (Figure 11), indicating that the evidence favouring THA over IF for reducing reoperation is conclusive and unlikely to be altered by future trials.

**Figure 12:**
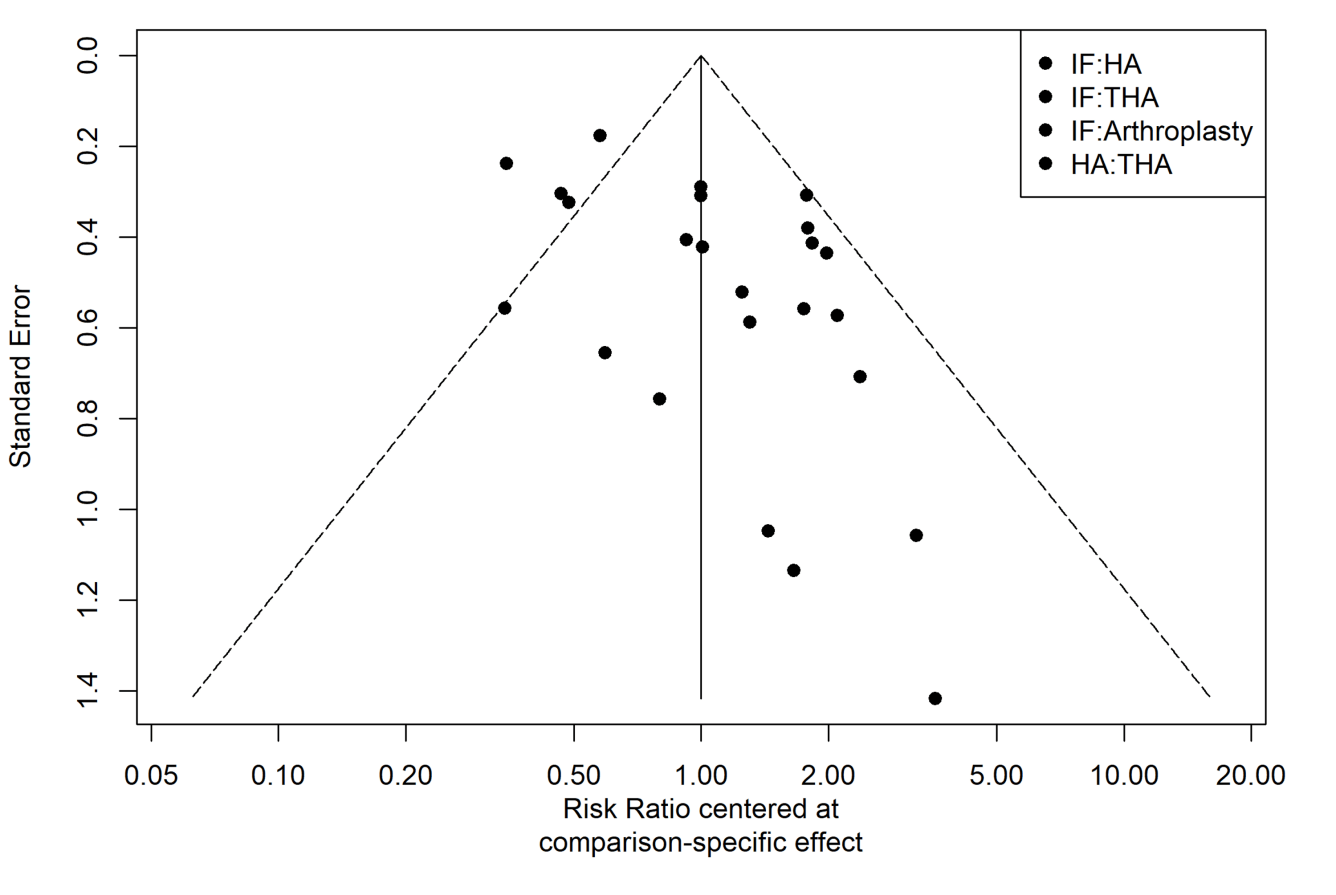
Comparison-Adjusted Funnel Plot Visual assessment of small-study effects and reporting bias for the reoperation outcome. IF, internal fixation; THA, total hip arthroplasty; HA, hemiarthroplasty

Comparison-adjusted funnel plots for the reoperation outcome revealed moderate asymmetry, which hints at the existence of small-study effects or possible publication bias (Figure 12). Egger’s test was statistically significant (p = 0.0094), indicating that smaller trials tended to report larger treatment effects favouring arthroplasty.

Certainty of Evidence (CINeMA)

The CINeMA assessment is summarized in Table 4. The certainty of evidence for THA vs. IF was graded as low due to major concerns regarding heterogeneity and global inconsistency. The comparison between HA and IF was graded as moderate, whereas THA versus HA reached moderate certainty, which was limited by imprecision in the effect estimates.

**Table 4:** CINeMA Evidence Profile for Surgical Interventions in Displaced FNF Downgrading primarily driven by inconsistency in the IF:THA comparisons and risk of bias in older trials. IF, internal fixation; THA, total hip arthroplasty; HA, hemiarthroplasty; CINeMA, confidence in network meta-analysis; FNF, femoral neck fracture

Comparison	Within-Study Bias	Reporting Bias	Indirectness	Imprecision	Heterogeneity	Incoherence	Overall Certainty
THA vs IF	Some concerns	No concerns	No concerns	Some concerns	Major concerns	No concerns	Low
HA vs IF	Some concerns	No concerns	No concerns	No concerns	Some concerns	No concerns	Moderate
THA vs HA	No concerns	No concerns	No concerns	Some concerns	No concerns	No concerns	Moderate

Discussion

This NMA, representing a comprehensive synthesis of 17 RCTs and 3,223 patients, provides robust evidence that primary arthroplasty, specifically THA, is superior to IF for the management of displaced FNFs in patients aged ≥65 years. The most critical finding is the reduction in the risk of reoperation associated with THA (RR 0.18) and HA (RR 0.35) compared to IF. Furthermore, the TSA confirms that the cumulative evidence has surpassed the RIS, establishing a definitive conclusion that is unlikely to be altered by future trials [34,41].

Arthroplasty Versus IF: Resolving Clinical Equipoise

The long-standing debate between joint-preserving IF and joint-replacing arthroplasty for displaced fractures has been settled by reoperation data. IF remains associated with unacceptably high failure rates, often exceeding 30%-40% in this age group, driven by nonunion and avascular necrosis [45,46,52]. In contrast, arthroplasty provides immediate skeletal stability and avoids the biological risks of impaired femoral head vascularity in displaced fractures [6,7]. While IF offers a shorter operative duration and reduced blood loss [8,9], these early perioperative benefits are overshadowed by the high clinical and economic burden of revision surgery. The SUCRA analysis identified THA as the most effective strategy for preventing secondary procedures (97.2%), supporting its role as a more definitive treatment option [33].

THA Versus HA

The comparative effectiveness of THA and HA remains a nuanced aspect of clinical decision-making. Although THA demonstrated superior functional outcomes at 24 months, with an improvement in HHS of 9.73 points over IF and 3.76 points over HA, the gain compared to HA did not reach the pre-defined seven-point threshold for an MCID [24,40,41]. Furthermore, while THA consistently exceeded the MCID threshold compared to IF, the 95% CI for HA (2.80-9.15) indicates that HA showed improvement that may or may not reach clinical significance depending on the individual patient. This suggests that while THA provides a statistically significant functional benefit, its clinical superiority over HA in the general geriatric population may be modest.

These functional gains must be balanced against the risk of postoperative instability. Although not reaching statistical significance in the pooled network estimates, THA reports higher dislocation rates in individual high-volume trials, such as the HEALTH study [41,56]. This risk is relevant in patients with cognitive impairment or neuromuscular disorders, who are frequently excluded from trials in this NMA [22,41]. For the “fit” and active elderly patient with high functional demands, THA remains the superior intervention. Conversely, for lower-demand or frailer patients, HA provides a reliable and technically less demanding alternative with stable functional restoration [2,6].

Methodological Strengths and Statistical Rigor

A primary strength of this study is the application of NMA, which allowed for a probabilistic ranking of all three interventions through the integration of direct and indirect evidence [25]. The use of the HKSJ adjustment ensures that the confidence intervals are robust and conservative, addressing the common issue of variance underestimation in random-effects models [27]. Furthermore, the TSA validates the conclusiveness of the reoperation results, providing a level of statistical certainty often absent in previous meta-analyses [11,34].

Limitations

We identified significant global and local inconsistencies within the reoperation network, particularly in the comparison between HA and IF. This statistical disagreement between direct and indirect evidence is driven by the broad chronological span of the included trials (1989-2019). Over these three decades, IF techniques evolved significantly (e.g., shifting from multiple cancellous screws to sliding hip screws), as did HA implant designs and postoperative protocols. Older trials comparing IF directly to HA may not perfectly align with the indirect estimates generated through modern THA trials. However, despite this local inconsistency, both direct and indirect estimates universally point toward the inferiority of IF. Therefore, while the precise magnitude of the risk reduction between HA and IF should be interpreted cautiously, the fundamental conclusion that arthroplasty safely minimizes reoperation compared to IF remains robust.

Furthermore, the result of Egger’s test (p = 0.0094) points to the possibility of small-study effects and publication bias. Visual inspection of the funnel plot reveals that smaller trials tended to report disproportionately larger treatment effects favoring arthroplasty. It must be acknowledged that this reporting bias may artificially inflate the apparent superiority of arthroplasty over IF in the pooled network estimates. The certainty of evidence for THA versus IF was graded as low via the CINeMA framework due to heterogeneity and inconsistency [38]. Finally, we lacked sufficient long-term data (>5 years) to evaluate the relative risks of late complications, such as acetabular erosion in HA or aseptic loosening in THA [5,12].

A further limitation is the inability to stratify outcomes by specific fracture severity. Most included trials reported Garden III and IV fractures cumulatively as “displaced” without providing extractable subgroup data. We could not evaluate whether the relative benefits of arthroplasty differ between partially displaced (Garden III) and completely displaced (Garden IV) fractures, the latter of which carries a higher risk of IF failure.

Clinical implications and future directions

The findings suggest that IF should be abandoned as a primary treatment for displaced FNFs in most patients aged >65 years, except in the most frail or terminal patients, in which minimizing surgical time is the sole priority. THA should be the preferred treatment for active patients, provided there are no significant contraindications to a more complex procedure. Future research should focus on refining the specific subgroups of the geriatric population that benefit most from THA, utilizing dual-mobility components to mitigate dislocation risks, and gathering robust long-term functional and patient-reported outcome data.

## Conclusions

Arthroplasty is the gold standard for displaced FNFs in patients aged ≥65 years. THA is the most effective surgical intervention for reducing the risk of reoperation and achieving optimal functional recovery. However, while THA is statistically superior to HA for functional restoration, this difference is likely not clinically noticeable for the average patient. Therefore, HA remains a highly effective and competitive option, particularly for frailer patients, balancing reliable functional outcomes with a lower risk of dislocation. The high failure rates of IF make it an inferior choice for this clinical population.
